# Microstructure, Mechanical and Tribological Properties of Arc Ion Plating NbN-Based Nanocomposite Films

**DOI:** 10.3390/nano12213909

**Published:** 2022-11-05

**Authors:** Yingying Fu, Hongxuan Li, Jianmin Chen, Hongjian Guo, Xiang Wang

**Affiliations:** 1 Bailie School of Petroleum Engineering, Lanzhou City University, Lanzhou 730070, China; 2 State Key Laboratory of Solid Lubrication, Lanzhou Institute of Chemical Physics, Chinese Academy of Sciences, Lanzhou 730000, China; 3 School of Bailie Mechanical Engineering, Lanzhou City University, Lanzhou 730070, China

**Keywords:** NbN/NbN-Ag film, nanocomposite film, microstructure, mechanical properties, tribological properties

## Abstract

NbN, NbN-Ag and NbN/NbN-Ag multilayer nanocomposite films were successfully deposited by an arc ion plating system (AIP), and their microstructures, mechanical and tribological properties were systematically investigated. The results show that all the films had a polycrystalline structure, and the Ag in the Ag-doped films existed independently as a face-centered cubic phase. The content of Ag in NbN-Ag and NbN/NbN-Ag films was 20.11 and 9.07 at.%, respectively. NbN films fabricated by AIP technique had excellent mechanical properties, and their hardness and critical load were up to 44 GPa and 34.6 N, respectively. The introduction of Ag into NbN films obviously reduced the friction coefficient at room temperature, while the mechanical properties and wear resistance were degraded sharply in comparison with that of NbN films. However, the NbN/NbN-Ag films presented better hardness, H/E*, *H*^3^/E*^2^, adhesive strength and wear resistance than NbN-Ag films. Additionally, analysis of wear surfaces of the studied films and Al_2_O_3_ balls using 3D images, depth profiles, energy dispersive spectrometry (EDS) and Raman spectra indicated that the main wear mechanisms of NbN and NbN/NbN-Ag films were adhesive and oxidation wear with slight abrasive wear, while the severe abrasive and oxidation wear were the dominant wear mechanism for NbN-Ag films.

## 1. Introduction

Transition metal nitride films (e.g., TiN, CrN, TiAlN, etc.) fabricated by different physical vapor deposition (PVD) techniques have been widely used for various purposes in the past few decades due to their superior mechanical properties, thermal stability, corrosion resistance and tribological properties [1,2,3,4,5,6,7]. However, with the rapid development of modern industry, more and more engineering components are utilized under harsher operating conditions of high speeds and high temperatures [8]; thus, the traditional nitride films are no longer effective in protecting workpieces due to their poor tribological properties at high-temperature.

In recent years, to meet the requirements for industrial development, several new nanocomposite films of MeN/Ag (Me = V, Mo, Nb, Ta, etc.) have been designed to improve tribological properties under high temperatures [9,10,11,12]. The NbN film has an ideal hard matrix due to its extremely high hardness (>40 GPa for the mixture of hexagonal β and δ′ phases) among the reported MeN films [13,14]. Additionally, numerous studies have shown that the soft noble metal of Ag possesses stable thermochemistry at mid–low temperature and good adhesion to the substrate surface [15]. Bondarev et al. illustrated that the Ag-doped films exhibited active oxidation protection and self-healing ability due to the segregation of metallic Ag particles at sites of cracking and oxidation [16,17]. The soft metal of Ag also has self-lubricating characteristics because of the lower shear strength of the Ag tribofilm generated on the wear surface during friction test at moderate temperatures [18,19,20]. Moreover, the introduction of Ag in NbN films is conductive to reducing friction at elevated testing temperatures, as the layered Nb_2_O_5_ and AgNbO_3_ can be formed on the sliding contact surfaces [21,22]. According to the above reasons, many studies have focused on the effect of silver content on the wear mechanisms of NbN-Ag films at elevated temperatures [21,23,24,25]. However, most of Ag-containing composite films exhibited a low friction coefficient only when the Ag content reached a higher level (e.g., about 18 at.% Ag even higher) [11,26], which is usually accompanied by a high wear rate at room temperature owing to the degradation of mechanical properties caused by Ag doping [23,24]. In fact the engineering components commonly undergo alternating conditions of room temperature at start-up and high-temperature stabilization stages. Therefore, it is essential to improve the mechanical properties and wear resistance of NbN-Ag composite films to prevent wear failure before reaching the high-temperature stabilization stage. The main purpose of this work is to improve the antiwear properties of Ag-doped films at room temperature.

In the current work, the NbN, NbN-Ag nanocomposite and NbN/NbN-Ag multilayer films were successfully fabricated by an arc ion plating system (AIP). Since the silver content can significantly affect the high-temperature tribological performance of NbN-Ag films, and the results of the experiment demonstrate that the films with 20.11 at.% Ag have good overall performance, especially the low friction coefficient at 600 °C. Therefore, the NbN-Ag film with Ag content of 20.11 at.% was selected for research. The NbN/NbN-Ag multilayer films were designed to obtain higher hardness and wear resistance in comparison to NbN-Ag composite films. Three kinds of multilayer films with different periodic thicknesses were deposited, among which only the multilayer films with the best friction properties were selected and compared with the NbN-Ag composite films. NbN films were also prepared as a contrast. The microstructure, mechanical and tribological properties were studied in detail, special attention was paid to the wear mechanism of these films at room temperature. The 3D images, depth profile, wear morphologies, chemical composition and Raman spectroscopy on wear surfaces of the films and the matched Al_2_O_3_ balls were analyzed to clarify the wear mechanism. 

## 2. Materials and Methods

### 2.1. Film Deposition

In this study, the NbN, NbN-Ag and NbN/NbN-Ag nanocomposite films were deposited on silicon (111) wafers and high-temperature alloy steel of GH4169 (JZSPC Inc., Suzhou, China) substrates using an AIP system. One niobium target (99.5%, Φ 100 mm) and one silver target (99.5%, Φ 100 mm) were employed as arc cathodes, which worked on mixed Ar (99.99%) and N_2_ (99.99%) gases. Prior to film deposition, the substrates were ultrasonically cleaned in deionized water, acetone and alcohol for 15 min successively, and then they were placed into the deposition chamber. After that, the chamber was heated up to 150 ℃; meanwhile, a background pressure of ~1 × 10^−3^ Pa was attained by the turbomolecular pumping system. Subsequently, the substrates were etched with Ar^+^ plasma generated by a DC unipolar pulse bias power supply (BPTC Inc., Beijing, China) in the chamber under a bias voltage of −800 V and a duty cycle of 70%, at a pressure of 1.4 Pa for 30 min, to remove the native oxides and contamination thereon. Then, in order to improve the bonding strength between the film and the substrate, a 120 nm thick Nb transition layer was prepared on the surface of the substrate with a target current of 135 A, a bias voltage of −250 V and an Ar flow rate of 220 sccm. The target current of Nb and the bias voltage on substrate were obtained using a DC power supply and a pulse bias power supply, respectively. Next, the ~150 nm thick transition layer was deposited by gradually decreasing the Ar flow rate from 220 to 65 sccm and increasing the nitrogen flow rate from 0 to 500 sccm to reduce the internal stress between the niobium interlayer and final films. Finally, the designed films were prepared on the top of the transition layer. The silver target was protected by shield cover during the deposition process of NbN film, while the shield cover was opened in the deposition process of the NbN-Ag film. The thickness of NbN and NbN-Ag layers in multilayer NbN/NbN-Ag nanocomposite films was controlled by the closing and opening times of the shield cover. The deposition time of each layer is 5 min. The preparation of studied nanocomposite films was performed in triplicate, and the obtained microstructures and performance could almost be completely repeated, regardless of the error allowed in the experiment. The detailed deposition parameters are listed in Table 1.

### 2.2. Characterization and Tribological Tests

The cross-sectional structure and surface morphology of the deposited nanocomposite films were studied by scanning electron microscopy (SEM; JEOL Inc., Tokyo, Japan). The chemical composition of the films was characterized by energy dispersive spectrometer (EDS). The microstructural studies on a nanoscale were performed by high-resolution transmission electron microscopy (HRTEM; FEI Inc., Hillsboro, FL, USA) and selected area electron diffraction (SAED) using FEI Tecnai G2 TF20 FE-TEM operated at 200 kV. The HRTEM samples of different film were prepared by focused ion beam (FIB, Ion source: Gallium liquid metal; Bruker Inc., Bremen, Germany). The film hardness was determined using a nano-indentation tester (CSM Inc., Locarno, Switzerland), and five repeated indentations were generated for each sample under same conditions. The penetration depth is 100 nm in all cases, which is less than 10% of film thickness. The adhesion strength between substrate and film was analyzed by a scratch tester (CSM Inc., Locarno, Switzerland) with the loading rate of 50 N/min, end load of 50 N and scratch length of 5 mm.

The tribological properties were evaluated in air atmosphere using a ball-on-disk tribometer (CSM Inc., Locarno, Switzerland) at RT. Al_2_O_3_ balls with diameter of 6 mm were used as counterparts. The sliding distance, average speed, normal load, relative humidity and the radius of the wear track were 100 m, 5 cm/s, 2 N, 35% and 5 mm, respectively. The friction tests were carried out three times for each film. Non-contact 3D surface profilometry (NANOVEA Inc., Irvine, CA, USA), the SEM and a Raman spectrometer (HORIBA Jobin Yvon Inc., Paris, France) were used to characterize the worn surfaces.

## 3. Results and Discussion

### 3.1. Microstructures and Mechanical Properties

**Figure 1:**
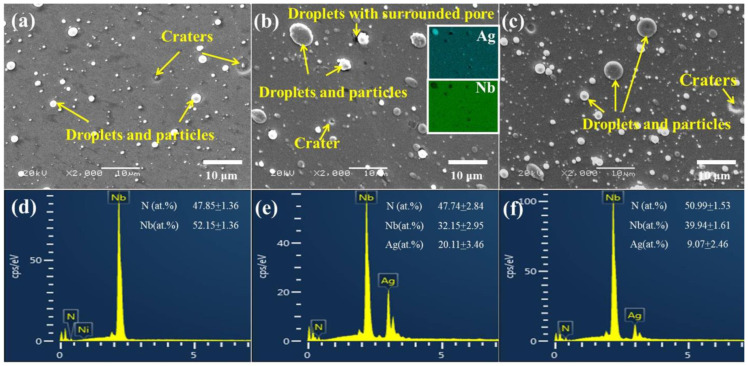
Surface morphologies and chemical compositions of the films: (**a**,**d**) NbN; (**b**,**e**) NbN-Ag; (**c**,**f**) NbN/NbN-Ag.

The typical surface morphologies and chemical compositions of the studied films are depicted in Figure 1. It is demonstrated that the as-deposited films have many surface defects, namely droplets, particles and craters, which are mainly caused by liquid droplets emitted from the surface of the Nb and Ag targets during film deposition process. The defect density on the surface of NbN-Ag and NbN/NbN-Ag films is higher than that of NbN film, which is due to the lower melting point of Ag (962 °C) than Nb (2468 °C), and hence the Ag droplets are formed more easily [27]. As can be seen from the insets of Figure 1b, the large Ag droplets and a relatively small Nb droplet are distributed on the Ag-doped film surface. The chemical composition of NbN film is close to stoichiometric ratio with 52.15 at.% Nb and 47.85 at.% N. The concentration of Ag in NbN-Ag and NbN/NbN-Ag multilayer nanocomposite films is 20.11 and 9.07 at.%, respectively. The error values of element contents in the NbN-Ag film are greater than those in NbN and multilayer films due to the influence of Ag droplets on the film surface.

**Figure 2:**
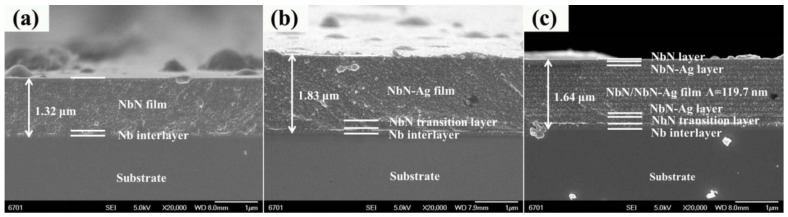
The FESEM cross-sectional images of the studied NbN-based nanocomposite films: (**a**) NbN; (**b**) NbN-Ag; (**c**) NbN/NbN-Ag.

Figure 2 shows the FESEM cross-sectional images of the resultant films. The thickness of the NbN, NbN-Ag and NbN/NbN-Ag films is 1.32, 1.83 and 1.64 μm, respectively. It is shown that all the studied films have both Nb interlayer and NbN transition layer. Here, the Nb interlayer plays an important role in improving the adhesion strength between substrates and films while the transition layer is designed to reduce the internal stress between the Nb interlayer and final films [28,29]. However, the interface between NbN transition layer and NbN film is indistinguishable (Figure 2a) due to their similar structure features. The cross-section of the NbN film exhibits a fish-scale-like structure, while that of NbN-Ag and NbN/NbN-Ag films (Figure 2b,c) shows a relatively smooth structure. Additionally, the NbN/NbN-Ag multilayer film consists of 12 NbN layers and 11 NbN-Ag layers, with a modulation period of ~119.7 nm, and a top layer of NbN.

**Figure 3:**
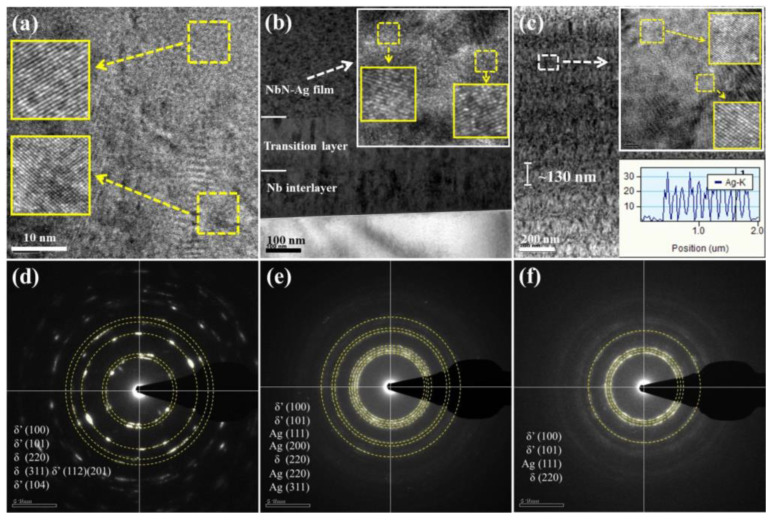
HRTEM cross-sectional micrographs and the corresponding SAED patterns of the films: (**a**,**d**) NbN; (**b**,**e**) NbN-Ag; (**c**,**f**) NbN/NbN-Ag.

The HRTEM cross-sectional micrographs and the corresponding SAED patterns of the studied films are shown in Figure 3. The crystal plane marked sequentially from top to bottom in Figure 3d–f corresponds to the radius of diffraction ring from small to large. Some locations on HRTEM micrograph were enlarged, as shown in the insets of Figure 3a, and distinct lattice fringes were clearly observed. It can be determined from the SAED patterns that the crystal planes of δ′ (100), (101), (112), (201) and (104) belong to the hexagonal close-packed (hcp) NbN phase, whilst the δ (220) and (311) crystal planes are assigned to the face-centered cubic (fcc) NbN phase (Figure 3d) [24]. The interfaces between the NbN transition layer and Nb interlayer, as well as the NbN-Ag film, are shown in Figure 3b. It is clear that there are no defects in the interfaces, suggesting the strong bonding between adjacent layers. Lattice fringes also appeared on the HRTEM micrograph of the NbN-Ag film, and the corresponding SAED pattern is presented in Figure 3e. The diffraction rings can be assigned to the crystal planes of (100), (101) for δ′-NbN, (220) for δ-NbN (111), (200) and (220) for fcc-Ag. Similar results were obtained in the previous report [21]. The cross-sectional HRTEM image of the multilayered NbN/NbN-Ag film is presented in Figure 3c, where the multilayer features of the film can be clearly seen. The modulation period thickness is ~130 nm, which is close to the value obtained by FESEM (Figure 2c). The insets show the periodic distribution of silver content along the direction of film growth and micrograph of multilayer film with distinguishable lattice fringes that revealed by diffraction rings corresponding to δ′-NbN (100), (101), δ-NbN (220) and fcc-Ag (111).

**Figure 4:**
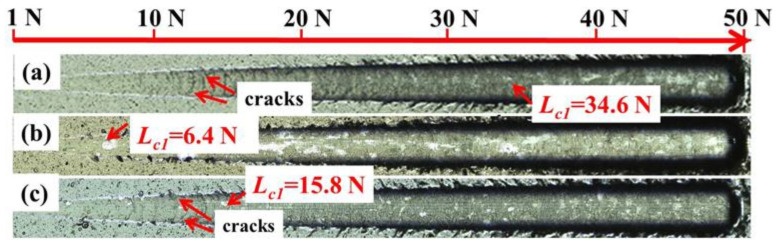
Optical microscope images of typical scratch morphologies of the films: (**a**) NbN; (**b**) NbN-Ag; (**c**) NbN/NbN-Ag.

Table 2 summarizes the hardness (*H*), reduced Young’s modulus (E*), *H*/E*, *H*^3^/E*^2^ and critical load (*L*_c1_, the load corresponding to first chipping) of the studied films. The *H* and E* of NbN film are 44.0 and 477.6 GPa, which are obviously higher than the NbN film deposited by magnetron sputtering technique recently [24], while the corresponding values of NbN-Ag film are 9.7 and 181.9 GPa, respectively. The NbN-Ag film has very low hardness compared with NbN film due to the fact that the Ag exists in a separate phase in the film according to the SAED analysis. It is worth mentioning that the hardness of NbN/NbN-Ag multilayer film is 19.8 GPa, and the value is greater than twice of NbN-Ag film. The results indicate that the hard NbN layer plays a significant role in improving hardness of multilayered film. According to the previous studies [30,31], the *H*/E* is an indicator of the resistance against elastic strain to failure of the film, and the *H*^3^/E*^2^ represents the resistance to plastic deformation of the film. The higher *H*^3^/E*^2^ values are often accompanied by higher *H*/E* values since some degree of plasticity needs high toughness [32], which is consistent with the results presented in Table 2. Generally, the higher H/E* and *H*^3^/E*^2^ ratios can endow the film with higher fracture toughness and hence good tribological performance [33]. The critical load (*L*_c1_) is 34.6 N for NbN film, 6.4 N for NbN-Ag film and 15.8 N for NbN/NbN-Ag film. To further observe the critical load points, the typical scratch morphologies tested by optical microscope are presented in Figure 4. The critical load of NbN film is as high as 34.6 GPa, and the characteristic of the film peeling off the substrate is too slight to discern. However, the film showed greater brittleness because cracks appeared prematurely during the scratch process (the load is about 12 N), which is attributing to the high hardness and Young’s modulus. The critical load of NbN-Ag film was only 6.4 N, and the debonding of the film was significantly increased in the subsequent test. The lower hardness of the film led to its rapid failure with the increase in the load. Nevertheless, the addition of Ag resulted in an enhanced toughness of the film, so there are no brittle fracture occurred [34]. Compared with NbN-Ag film, multilayer film has good bearing capacity due to its high hardness, and thus, the critical load value is higher and no serious peeling in the whole test. The strong adhesion also plays a positive role in improving film tribological properties. 

### 3.2. Frictional Wear Properties and Wear Mechanisms

**Figure 5:**
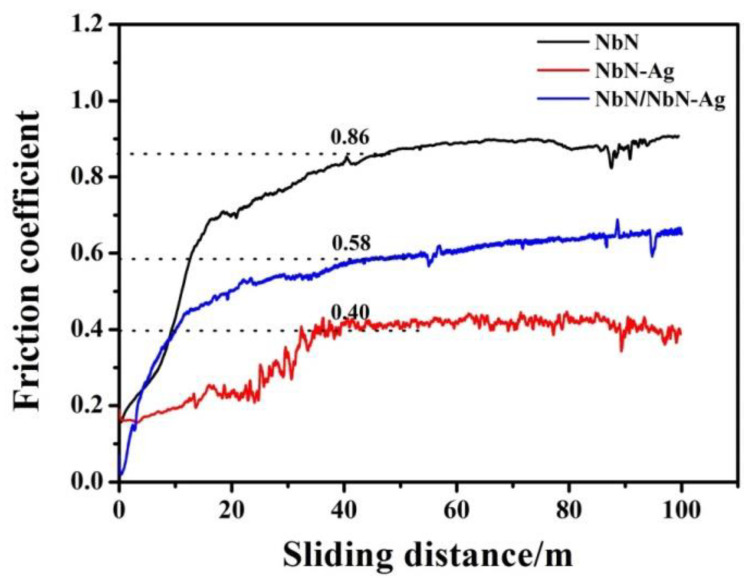
Friction coefficient curves of studied films sliding against Al_2_O_3_ balls at room temperature.

**Figure 6:**
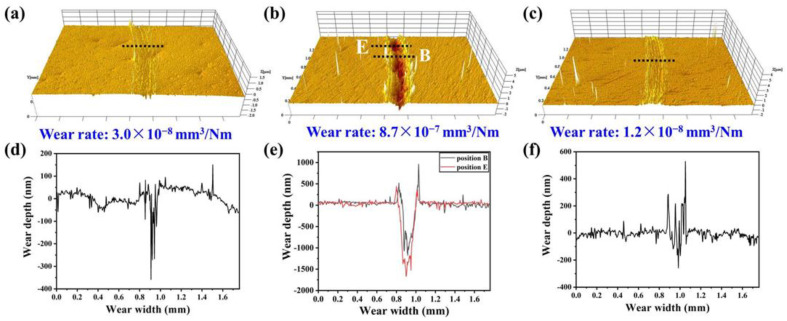
3D images and depth profile of worn surfaces for studied films: (**a**,**d**) NbN; (**b**,**e**) NbN-Ag; (**c**,**f**) NbN/NbN-Ag.

In Figure 5 the tribological tests for the studied films are reported, where the friction coefficient of the films sliding against Al_2_O_3_ balls at room temperature is plotted as a function of sliding distance. The friction coefficients of the NbN and NbN/NbN-Ag films increase sharply in the first 15 m and then increase very slightly in the following test. The friction coefficient of NbN-Ag film increases in the first 37 m and then it almost keeps constant during the remaining test period with minor fluctuation. The average friction coefficient of NbN/NbN-Ag film is about 0.58, while a relatively high value of 0.86 is observed for NbN film. The lowest friction coefficient about 0.40 is obtained for NbN-Ag film. The 3D images and depth profiles of the worn surfaces for the studied films are displayed in Figure 6. The worn surfaces of NbN and NbN/NbN-Ag films are relatively smooth except for some shallow furrows, while the NbN-Ag film has a relatively rough worn surface. The average wear depths of NbN and NbN/NbN-Ag films are about 200 and 180 nm while the wear rate of that films are 3.0 × 10^−8^ and 1.2 × 10^−8^ mm^3^/Nm, respectively. The wear depth of position *B* and *E* on wear track of NbN-Ag film is respective 1600 and 1100 nm, and the average wear rate is 8.7 × 10^−7^ mm^3^/Nm. The lower wear rates, characteristics of shallow furrows and wear profiles of the worn surfaces show that the NbN and the NbN/NbN-Ag multilayer film suffered slight abrasive wear. The wear depth of NbN-Ag film is close to the film thickness indicating a severe abrasive wear occurred, and thus, a high wear rate of 8.7 × 10^−7^ mm^3^/Nm is achieved. In addition, it is found that the two sides of wear tracks for NbN-Ag and NbN/NbN-Ag films are higher than the original film surface to some extent, implying that the plastic deformation took place during friction period.

**Figure 7:**
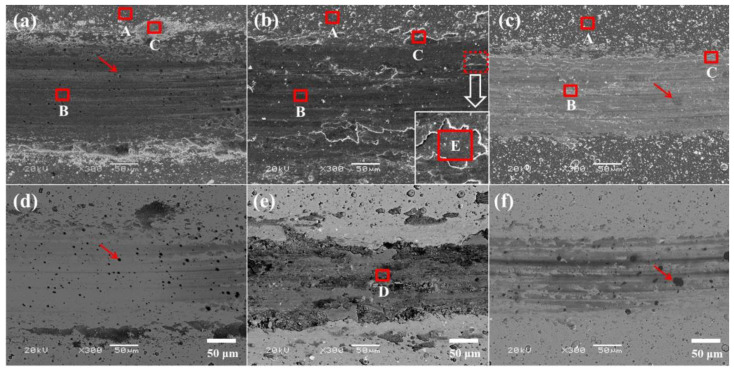
SEM and back-scattering images of worn surfaces for studied films: (**a**,**d**) NbN; (**b**,**e**) NbN-Ag; (**c**,**f**) NbN/NbN-Ag.

The typical worn surfaces of the studied films are presented in Figure 7. The chemical compositions of different regions on worn surfaces (marked by A, B, C, D and E in Figure 7) were determined by EDS, and the final results are summarized in Table 3. Here, letter A represents of the regions near the wear tracks, while letters B, D and E stand for the regions in the middle of wear tracks, and C represents the regions on the edge of the wear tracks. For NbN film, the chemical composition (at.%) in region A is very close to the original film. Region B includes 47.14 at.% Nb, 31.35 at.% N and 21.51 at.% O, and the high oxygen content indicates that the worn surface was partially oxidized during the friction period. Combined with Figure 7 and Table 3, it can be inferred that region C is covered by wear debris of both niobium oxides and alumina. Additionally, some black dots in the wear track (indicated by arrows) are wear debris of the Al_2_O_3_ balls, which is confirmed by EDS analysis. For NbN-Ag film, the wear track is rougher compared with NbN film, and it seems that the wear debris was rolled by the mated ball and adhered to the wear track (Figure 7b). The Ag content in region A (21.84 at.%) is higher than that of the original film (20.11 at.%); this can be attributed to the error of EDS method, which is mainly caused by the presence of large Ag particles on the surface of the NbN-Ag film (see the inset of Figure 1). A small amount of oxygen (3.60 at.%) is also present in region A, and this is attributed to the slight oxidation of the film. Regions B and E inside the wear track correspond to positions B and E, as shown in Figure 6b, respectively. Region B contains a small amount of N, and the concentration of some substrate elements such as Ni, Cr and Fe in region E is higher than that in region B, illustrating that the film has been partially worn out. This is in good agreement with the results obtained by 3D morphology and the depth profiles of the worn surface. A certain amount of oxygen (23.32 at.% in region B and 51.71 at.% in region E) is present in the wear track, which is due to the serious oxidation of the sliding surface. Although the features of B and D in the wear track are different from each other, the chemical compositions of both regions are similar. Due to the presence of partially oxidized wear debris in region C, 26.19 at.% O and 2.02 at.% Ni were detected in this region. The worn surface of the NbN/NbN-Ag film is negligible, and there is no obvious wear debris scattered along the edges of the wear track (Figure 7f). It can be seen from Table 3 that the oxygen content in regions B and C is relatively high for this film, indicating that the film is severely oxidized in the sliding wear regions. Additionally, the Ag content in the regions of A, B and C is slightly higher than that of original film (9.07 at.%), but within the error value. The wear debris of the Al_2_O_3_ balls, as indicated by arrows, are also found in the regions B and C.

**Figure 8:**
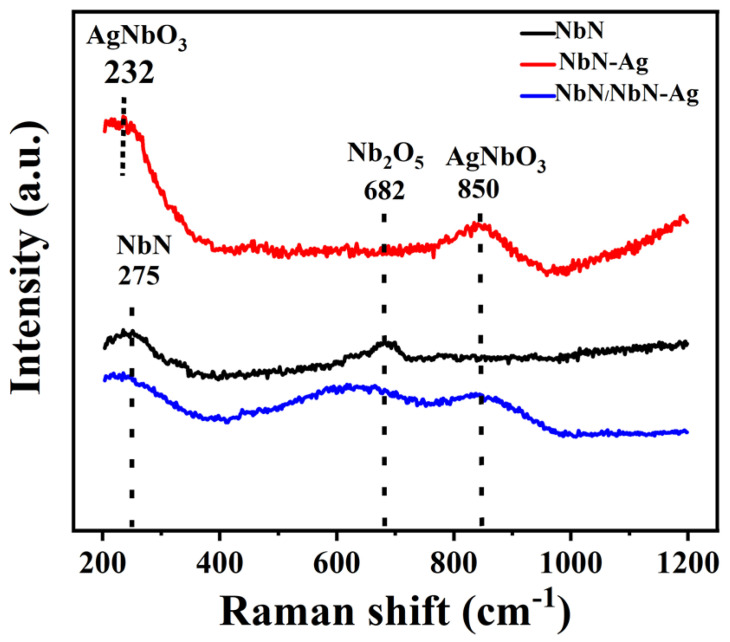
Raman spectra of the wear tracks of the NbN, NbN-Ag and multilayer films after friction test at RT.

To investigate the wear products, the Raman spectra obtained from the wear tracks of the studied films are shown in Figure 8. The Raman spectrum of the wear track of the pure NbN film consists of two peaks at 275 and 682 cm^−1^ corresponding to NbN and Nb_2_ O_5_ [21,22]. It is demonstrated that the worn surface of NbN film is partially oxidized, which is consistent with results of EDS analysis. Two peaks of NbN at 275 and AgNbO_3_ at 850 cm^−1^ for NbN-Ag film were observed [22]. This suggests that the Ag added NbN film can form the lubricant of AgNbO_3_ on the wear track during friction test. The oxidation products of tribological test of multilayer film included Nb_2_O_5_ and AgNbO_3_. The intensity of the NbN peak at 275 is very weak, implying that the wear surface of the film suffered severe oxidation. The generation of Nb_2_O_5_ and AgNbO_3_ by tribochemical reactions can significantly reduce the friction, therefore, the low friction coefficients of Ag-doped films were achieved, as shown in Figure 5.

**Figure 9:**
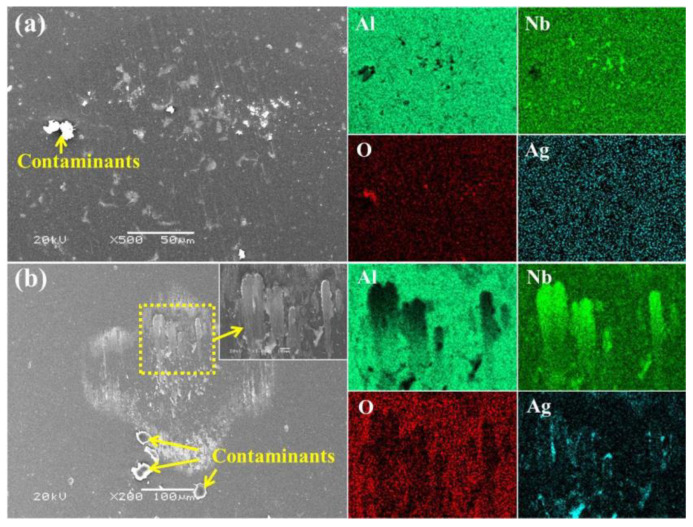
Wear scars for Al_2_O_3_ balls after sliding against NbN film (**a**) and NbN-Ag film (**b**), as well as corresponding EDS maps.

**Figure 10:**
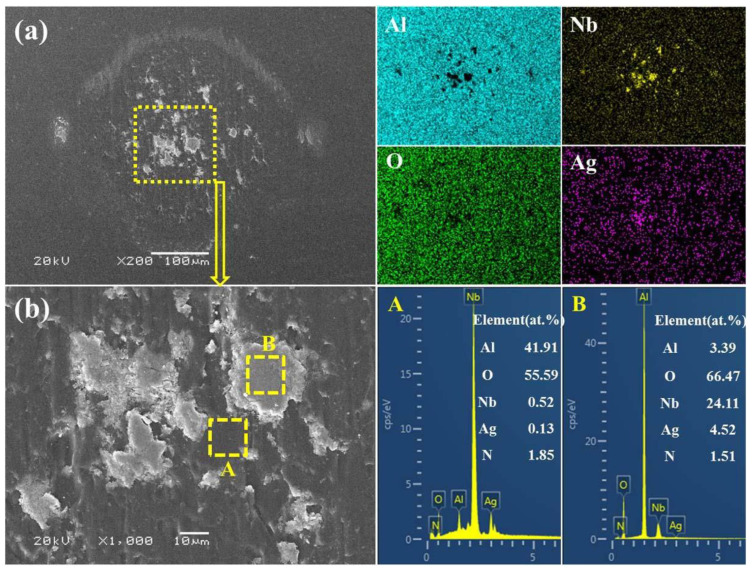
Wear scar for Al_2_O_3_ ball after sliding against NbN/NbN-Ag film (**a**) and its magnified image (**b**), as well as corresponding EDS maps of (**a**) and the EDS spectra for selected regions A and B in (**b**).

The worn surfaces of Al_2_O_3_ balls were also investigated by SEM and corresponding EDS maps after the balls were ultrasonically cleaned in deionized water, and the results are demonstrated in Figure 9 and Figure 10. It can be seen from Figure 9a that the Al_2_O_3_ ball experienced slight abrasive wear when it slid against the NbN film, and no obvious wear scar was found even by SEM. The elements distribution of EDS maps shows some small Nb-rich regions in the middle of the wear scar, indicating that slight adhesive wear occurred. The mated ball sliding against NbN-Ag film has a definite wear scar (see Figure 9b), and some lamellar adhesive materials are observed at the center of wear scar (see the inset in Figure 9b). The element distribution demonstrates that the adhered materials are rich in Nb and Ag, which suggests the main wear mechanism is adhesive wear. In Figure 10 the wear scar of the Al_2_O_3_ ball after sliding against NbN/NbN-Ag film is relatively smooth and some transferred materials with high Nb and Ag content are observed in the middle of wear scar, suggesting adhesive wear also occurred during the friction process. The magnified image of dotted box in Figure 10a, and the chemical composition of regions A and B in Figure 10b are also provided. In comparison with the chemical composition in region A, it is not difficult to determine that the adhered material of region B is also mainly composed of Nb, Ag and O elements. Therefore, it can be confirmed that the bonding layer in region B is mainly composed of Nb and Ag oxides, which is consistent with the results reported in the literature [24,35].

According to the above tests and characterization, the friction and wear mechanisms of the studied films are described as follows. The wear loss of NbN film and mated balls during the running-in period generated a small amount of hard wear debris principally consisting of Nb_2_O_5_ and NbN, part of which was dispersed at the edges of wear tracks, and other parts were entrapped in the sliding interfaces. The entrapped wear debris would further plow the sliding interfaces or become rolled by the mated ball during the next friction period, leading to the formation of furrows and some Al_2_O_3_ wear debris adhered to the wear track. As a result of this, a high friction coefficient about 0.86 is obtained. However, the slight wear of NbN film is due to its high *H*, H/E* and H^3^/E*^2^ values since they can endow the nanocomposite film with high wear resistance [31]. The characteristics of shallow furrows (see Figure 6a,d, Figure 7a,d and Figure 9a) and the elemental composition (see Table 3 and EDS maps of Figure 9a) of wear surfaces of films and the matched Al_2_O_3_ balls suggest that the wear mechanisms of NbN films were oxidation wear together with slight abrasive wear, along with a low wear rate of 3.0 × 10^−8^ mm^3^/Nm. The wear debris of NbN-Ag film was easily formed on the sliding surfaces at the beginning of friction due to the low hardness of this film relative to the mated Al_2_O_3_ ball. The soft wear debris was mainly composed of AgNbO_3_ formed by tribochemical reactions during the friction process. The product of AgNbO_3_ was entrapped between sliding interfaces and adhered to the parent worn surface (see Figure 9b), leading to the formation of a discontinuous transfer layer on the worn surface. This transfer layer of AgNbO_3_ partially separated the mated ball and the film surface, and hence low friction was obtained. Thus, the main wear mechanism of NbN-Ag films was serious abrasive wear, which is ascribed to the low critical load and low hardness. Moreover, the large amount of oxygen (see Table 3) on the wear surface and the AgNbO_3_ detected by the Raman method indicates the occurrence of oxidation wear for the NbN-Ag film. As a result, the NbN-Ag film presented a high wear rate. The mechanical properties of NbN-Ag nanocomposite film are greatly improved via designing the NbN/NbN-Ag multilayer structure. Generally speaking, the wear resistance is strongly dependent upon the film hardness, and the increased hardness could reduce the real contact region with the matched ball [3], and thus, the hard NbN/NbN-Ag multilayer film exhibited superior wear resistance compared with the soft NbN-Ag nanocomposite film. The predominant wear mechanisms for multilayer film were adhesive wear and oxidation wear, since the Nb, Ag and O elements of film were found on the wear scars of Al_2_O_3_ balls (see Figure 10), and Nb_2_O_5_ as well as AgNbO_3_ existed on the wear track of multilayer film. Although the friction coefficient of NbN/NbN-Ag multilayer film is 0.58, which is slightly higher than that of NbN-Ag nanocomposite film, ~0.40, the wear resistance of multilayer film is obviously higher than that of the nanocomposite films, and thus, a low wear rate was obtained. 

It is commonly known that the coalescence of silver and the generation of metal oxides of Nb_2_O_5_ and AgNbO_3_ on the wear tracks of the Ag-doped NbN films during tribological period could act as effective lubricants at elevated temperatures [12,24]. Therefore, controlling the diffusion rate of silver from the film interior to the wear surface is likely to prolong the wear life of the NbN-Ag film. The NbN layer is expected to play a role as the diffusion barrier of Ag in NbN/NbN-Ag multilayer films, similar to the CrN layer on CrN-Ag film and the TiN layer on YSZ–Ag–Mo-based nanocomposite coatings [36,37]. Thus, the NbN/NbN-Ag multilayer film could be a promising alternative for NbN-Ag film in terms of antifriction and wear, and finally prolong the service life of the film both at room and elevated temperatures.

## 4. Conclusions

Three kinds of NbN-based nanocomposite films were successfully prepared using the AIP system, and the microstructures and the mechanical and tribological properties of the resultant films were systematically investigated using advanced characterization techniques. The main conclusions can be drawn as follows:

(1) The NbN film was stoichiometric, while the contents of Ag in the NbN-Ag and NbN/NbN-Ag films were 20.11 and 9.07 at.%, respectively. The NbN film exhibited superior mechanical properties, while the NbN-Ag film had low hardness and poor adhesive strength due to the fact that the Ag existed in a separate phase in the film interior and possessed low hardness. However, the mechanical properties of NbN-Ag film were significantly improved by designing the NbN/NbN-Ag multilayer structure.
(2) The introduction of Ag into the NbN film can effectively reduce the friction coefficient but significantly increase the wear loss. In contrast to the NbN-Ag film, the higher friction and lower wear loss of NbN/NbN-Ag film were mainly ascribed to its relatively lower Ag content and superior mechanical properties, respectively. The results show that the main wear mechanisms of NbN and NbN/NbN-Ag films were adhesive and oxidation wear with slight abrasive wear, while the severe abrasive and oxidation wear dominated the wear mechanism of NbN-Ag films.
(3) The NbN/NbN-Ag multilayer film could be a promising alternative for NbN-Ag film to prolong the service life of the film both at room and elevated temperatures, depending on its excellent mechanical properties, wear resistance and excellent lubrication effect of Nb_2_O_5_ and AgNbO_3_.

## Figures and Tables

**Table 1:** The deposition parameters of the studied films.

Parameters		Value
Working pressure (Pa)		0.42
Chamber temperature (°C)		150
Ar:N_2_ ratio (sccm)		65:500
Duty cycle (%)		40
Nb target current (A)		135
Ag target current (A)		50
Negative dc pulse bias (−V)		200
Deposition time (min)	NbN	110
	NbN-Ag	105
	NbN/NbN-Ag	110 (5/5)

**Table 2:** The mechanical performances of the studied films.

Mechanical properties	NbN	NbN-Ag	NbN/NbN-Ag
Hardness (*H*, GPa)	44.0	9.7	19.8
Elastic modulus (E*, GPa)	477.6	181.9	291.3
H/E*	0.092	0.053	0.068
*H*^3^/E*^2^ (GPa)	0.372	0.027	0.092
Critical load (*L*_c1_, N)	34.6	6.4	15.8

**Table 3:** The EDS analysis results for different positions in Figure 7.

Film	NbN			NbN-Ag					NbN/NbN-Ag		
Region	A	B	C	A	B	C	D	E	A	B	C
Nb (at.%)	50.04 ± 1.32	47.14 ± 1.39	44.61 ± 1.89	29.19 ± 1.33	42.68 ± 2.02	39.32 ± 1.47	44.02 ± 1.67	10.10 ± 1.85	43.22 ± 1.73	45.32 ± 1.82	51.34 ± 1.33
N (at.%)	49.96 ± 1.32	31.35 ± 1.47	-	45.37 ± 1.46	7.87 ± 1.57	9.01 ± 1.51	6.38 ± 2.10	-	47.10 ± 2.33	0.95 ± 0.51	1.02 ± 0.55
Ag (at.%)	-	-	-	21.84 ± 1.22	10.12 ± 1.63	23.46 ± 2.01	11.89 ± 1.67	-	9.68 ± 2.18	9.19 ± 1.42	11.02 ± 1.10
O (at.%)	-	21.51 ± 1.61	54.63 ± 1.34	3.60 ± 1.21	23.32 ± 1.56	26.19 ± 1.28	20.82 ± 1.28	51.71 ± 2.24	-	43.10 ± 1.09	35.41 ± 2.10
Al (at.%)	-	-	0.76 ± 1.51	-	-	-	-	-	-	1.44 ± 0.76	1.21 ± 0.77
Cr (at.%)	-	-	-	-	3.51 ± 1.22	-	4.05 ± 0.91	8.78 ± 1.77	-	-	-
Fe (at.%)	-	-	-	-	3.64 ± 1.01	-	3.86 ± 0.87	8.95 ± 1.68	-	-	-
Ni (at.%)	-	-	-	-	8.86 ± 1.79	2.02 ± 1.36	8.98 ± 1.66	20.46 ± 1.45	-	-	-

## Data Availability

Data are available from Y.F. upon reasonable request.

## References

[1] Ou Y.X., Lin J., Tong S., Che H.L., Sproul W.D., Lei M.K. (2015). Wear and corrosion resistance of CrN/TiN superlattice coatings deposited by a combined deep oscillation magnetron sputtering and pulsed dc magnetron sputtering. Appl. Surf. Sci..

[2] Wu Z., Zhou F., Chen K., Wang Q., Zhou Z., Yan J., Li L.K.-Y. (2016). Friction and wear properties of CrSiCN coatings with low carbon content as sliding against SiC and steel balls in water. Tribol. Int..

[3] Melnikova G., Kuznetsova T., Lapitskaya V., Petrovskaya A., Chizhik S., Zykova A., Safonov V., Aizikovich S., Sadyrin E., Sun W. (2021). Nanomechanical and nanotribological properties of nanostructured coatings of tantalum and Its compounds on steel substrates. Nanomaterials.

[4] Podgornik B., Sedlaček M., Mandrino D. (2016). Performance of CrN coatings under boundary lubrication. Tribol. Int..

[5] Chen L., Ran Y., Jiang Z., Li Y., Wang Z. (2020). Structural, compositional, and plasmonic characteristics of Ti–Zr ternary nitride thin films tuned by the nitrogen flow ratio in magnetron sputtering. Nanomaterials.

[6] Zhang Z., Zhang L., Yuan H., Qiu M., Zhang X., Liao B., Zhang F., Ouyang X. (2022). Tribological behaviors of super-hard TiAlN coatings deposited by filtered cathode vacuum arc deposition. Materials.

[7] Zin V., Montagner F., Deambrosis S.M., Mortalò C., Litti L., Meneghetti M., Miorin E. (2022). Mechanical and tribological properties of Ta-N and Ta-Al-N coatings deposited by reactive high power impulse magnetron sputtering. Materials.

[8] Musil J. (2000). Hard and superhard nanocomposite coatings. Surf. Coat. Technol..

[9] Aouadi S.M., Paudel Y., Luster B., Stadler S., Kohli P., Muratore C., Hager C., Voevodin A.A. (2008). Adaptive Mo_2_N/MoS_2_/Ag Tribological Nanocomposite Coatings for Aerospace Applications. Tribol. Lett..

[10] Chen F., Feng Y., Shao H., Zhang X., Chen J., Chen N. (2012). Friction and Wear Behaviors of Ag/MoS_2_/G Composite in Different Atmospheres and at Different Temperatures. Tribol. Lett..

[11] Aouadi S.M., Singh D.P., Stone D.S., Polychronopoulou K., Nahif F., Rebholz C., Muratore C., Voevodin A.A. (2010). Adaptive VN/Ag nanocomposite coatings with lubricious behavior from 25 to 1000 °C. Acta Mater..

[12] Stone D.S., Migas J., Martini A., Smith T., Muratore C., Voevodin A.A., Aouadi S.M. (2012). Adaptive NbN/Ag coatings for high temperature tribological applications. Surf. Coat. Technol..

[13] Sandu C.S., Benkahoul M., Parlinska-Wojtan M., Sanjinés R., Lévy F. (2006). Morphological, structural and mechanical properties of NbN thin films deposited by reactive magnetron sputtering. Surf. Coat. Technol..

[14] Bendavid A., Martin P.J., Kinder T.J., Preston E.W. (2003). The deposition of NbN and NbC thin films by filtered vacuum cathodic arc deposition. Surf. Coat. Technol..

[15] Mulligan C.P., Blanchet T.A., Gall D. (2008). CrN–Ag nanocomposite coatings: Effect of growth temperature on the microstructure. Surf. Coat. Technol..

[16] Bondarev A.V., Kiryukhantsev-Korneev P.V., Levashov E.A., Shtansky D.V. (2017). Tribological behavior and self-healing functionality of TiNbCN-Ag coatings in wide temperature range. Appl. Surf. Sci..

[17] Voevodin A., Zabinski J. (2005). Nanocomposite and nanostructured tribological materials for space applications. Compos. Sci. Technol..

[18] Hsieh J.H., Chiu C.H., Li C., Wu W., Chang S.Y. (2013). Development of anti-wear and anti-bacteria TaN-(Ag,Cu) thin films—A review. Surf. Coat. Technol..

[19] Wu Y., Chen J., Li H., Ji L., Ye Y., Zhou H. (2013). Preparation and properties of Ag/DLC nanocomposite films fabricated by unbalanced magnetron sputtering. Appl. Surf. Sci..

[20] Bondarev A.V., Kiryukhantsev-Korneev P.V., Sidorenko D.A., Shtansky D.V. (2016). A new insight into hard low friction MoCN–Ag coatings intended for applications in wide temperature range. Mater. Des..

[21] Ju H., Xu J. (2015). Microstructure and tribological properties of NbN-Ag composite films by reactive magnetron sputtering. Appl. Surf. Sci..

[22] Ren P., Zhang K., He X., Du S., Yang X., An T., Wen M., Zheng W. (2017). Toughness enhancement and tribochemistry of the Nb-Ag-N films actuated by solute Ag. Acta Mater..

[23] Ju H., Ding N., Xu J., Yu L., Geng Y., Ahmed F. (2019). The tribological behavior of niobium nitride and silver composite films at elevated testing temperatures. Mater. Chem. Phys..

[24] Ren Y., Jia J., Cao X., Zhang G., Ding Q. (2022). Effect of Ag contents on the microstructure and tribological behaviors of NbN–Ag coatings at elevated temperatures. Vacuum.

[25] Dai X., Wen M., Wang J., Cui X., Wang X., Zhang K. (2020). The tribological performance at elevated temperatures of MoNbN-Ag coatings. Surf. Coat. Technol..

[26] Mulligan C.P., Gall D. (2005). CrN–Ag self-lubricating hard coatings. Surf. Coat. Technol..

[27] Smyrnova K., Sahul M., Harsani M., Pogrebnjak A., Ivashchenko V., Beresnev V., Stolbovoy V., Caplovic L., Caplovicova M., Vanco L. (2022). Microstructure, mechanical and tribological properties of advanced layered WN/MeN (Me = Zr, Cr, Mo, Nb) nanocomposite coatings. Nanomaterials.

[28] Xu F., Xu J.H., Yuen M.F., Zheng L., Lu W.Z., Zuo D.W. (2013). Adhesion improvement of diamond coatings on cemented carbide with high cobalt content using PVD interlayer. Diam. Relat. Mater..

[29] Singh K., Bidaye A.C., Suri A.K. (2011). Magnetron Sputtered NbN Films with Nb Interlayer on Mild Steel. Int. J. Corros..

[30] Lin J., Moore J.J., Mishra B., Pinkas M., Zhang X., Sproul W.D. (2009). CrN/AlN superlattice coatings synthesized by pulsed closed field unbalanced magnetron sputtering with different CrN layer thicknesses. Thin Solid Films.

[31] Leyland A., Matthews A. (2000). On the significance of the H/E ratio in wear control: A nanocomposite coating approach to optimised tribological behaviour. Wear.

[32] Wang C., Shi K., Gross C., Pureza J.M., de Mesquita Lacerda M., Chung Y.-W. (2014). Toughness enhancement of nanostructured hard coatings: Design strategies and toughness measurement techniques. Surf. Coat. Technol..

[33] Guan X., Wang Y., Zhang G., Jiang X., Wang L., Xue Q. (2017). Microstructures and properties of Zr/CrN multilayer coatings fabricated by multi-arc ion plating. Tribol. Int..

[34] Bondarev A.V., Golizadeh M., Shvyndina N.V., Shchetinin I.V., Shtansky D.V. (2017). Microstructure, mechanical, and tribological properties of Ag-free and Ag-doped VCN coatings. Surf. Coat. Technol..

[35] Wu F., Yu L., Ju H., Asempah I., Xu J. (2018). Structural, mechanical and tribological properties of NbCN-Ag nanocomposite films deposited by reactive magnetron Sputtering. Coatings.

[36] Hu J.J., Muratore C., Voevodin A.A. (2007). Silver diffusion and high-temperature lubrication mechanisms of YSZ–Ag–Mo based nanocomposite coatings. Compos. Sci. Technol..

[37] Mulligan C.P., Blanchet T.A., Gall D. (2010). Control of lubricant transport by a CrN diffusion barrier layer during high-temperature sliding of a CrN–Ag composite coating. Surf. Coat. Technol..

